# Joint Session Central Texas and Austin District Medical Societies

**Published:** 1892-09

**Authors:** W. R. Blailock, W. O. Wilkes

**Affiliations:** President; Secretary


### Society Notes.

### JOINT SESSION.

There will be a joint session of the Austin District Medical Society, and the Central Texas Medical Association, held at Waco, Texas, Tuesday, October 11th, 1892. Following is the announcement and programme:

"DEAR DOCTOR—In your professional work do you not sometimes realize that new light has shed its radiance upon, and is illuminating some fact connected with human ills that was previously dark and obscure to you? On these occasions, do you not feel an instinctive impulse to seek a professional brother and communicate it to him?

"On Tuesday, October 11th, 1892, at Waco, Texas, there will be an assembling of your brother physicians, who will have something to impart to you, and who will be pleased to have you impart something to them, in regard to the alleviation and cure of human ills.

"This being the twenty-fifth quarterly session of the Central Texas Medical Association, and a joint session with the Austin District Medical Society, it requires no stretch of the imagination to predict one of the most interesting, profitable and enjoyable sessions in the history of the Association.

"We will be glad to have you meet with us, join in the discussion, present a paper, and report interesting cases from private practice. W. R. BLAILOCK, President.

"W. O. WILKES, Secretary."

---

PROGRAMME.

1. "Gastro-entero-colitis of Children," by Dr. G. W. Christian, of Burnet. Discussion to be opened by Dr. J. W. McLaughlin and Dr. T. J. Bennett, of Austin.

2. "Typhoid Fever—Etiology, Pathology, Differential Diagnosis and Treatment," by Dr. W. R. Blailock, of McGregor. Discussion to be opened by Dr. A. H. Snead and Dr. W. H. Wilkes, of Waco.

3. "Enlargement of the Prostate," by Dr. A. N. Denton, of Austin. Discussion to be opened by Dr. B. E. Hadra, of Galveston, and Dr. T. D. Wooten, of Austin.

4. "Syphilis—Its Prevention and Cure," by Dr. W. E. Brown, of Gatesville. Discussion to be opened by Dr. M. L. Graves, of Waco, and Dr. J. M. Woodson, of Temple.

5. Volunteer Papers.

6. Reports of Cases.

Banquet at night.

---

By order of the council, the annual meeting of the Southern Surgical and Gynecological Association has been postponed from the 8th, 9th and 10th, until the 15th, 16th and 17th of November. It was thought wise to change the time of the meeting from the fact that the 8th of November is the date of the Presidential election. Everything points to a very successful session. So states W. E. B. Davis, M. D., Secretary.

